# Preparation and Optimization of a Polyhydroxyoctanoate–Hydroxyapatite Composite Available to Scaffolds in Implantable Devices

**DOI:** 10.3390/molecules30030730

**Published:** 2025-02-06

**Authors:** Dana-Maria Miu, Ramona Daniela Pavaloiu, Fawzia Sha’at, Mariana-Gratiela Vladu, Georgeta Neagu, Vasile-Sorin Manoiu, Mihaela-Carmen Eremia

**Affiliations:** 1 National Institute of Chemical-Pharmaceutical Research and Development-ICCF, 112 Vitan Avenue, 3rd District, 031299 Bucharest, Romania; 2 Faculty of Chemical Engineering and Biotechnologies, National University of Science and Technology Politehnica, 1 Gheroghe Polizu Street, 1st District, 011061 Bucharest, Romania; 3 National Institute of Research and Development for Biological Sciences, 296 Spaiul Independentei Street, 6th District, 060031 Bucharest, Romania

**Keywords:** polyhydroxyalkanoates, hydroxyapatite, composites, materials, biomedical

## Abstract

Biomaterials represent a distinct class of materials used in various medical applications, such as replicating the shape or function of damaged tissue caused by disease or trauma. The increasing focus on polyhydroxyalkanoate (PHA) research can be attributed to their properties, such as biodegradability, biocompatibility, and bioresorbability. PHAs can be incorporated into polymeric complexes or combined with bioceramics or bioactive substances. Films of PHO-HAp-Curcumin were prepared, and optimization studies were conducted using Design-Expert software (Stat-Ease 360-Trial Version). The effects of independent variables (amount of PHO, HAp, and curcumin) on biodegradability, film thickness, and curcumin release were studied. Statistical modeling revealed significant interactions among the components, with the 2FI and quadratic models providing strong predictive accuracy. The interaction of HAp and PHO amounts (X_2_X_3_) has a significant effect on biodegradability (Y_1_) and film thickness (Y_3_). For the degree of the cumulative release of curcumin (CDR), there was no significant interaction between the independent variables (curcumin-X_1_, HAp-X_2_, and PHO-X_3_). Optimized film exhibited a maximum desirability of 0.777 with 1 mg of curcumin, 100 mg of HAp, and 172.31 mg of PHO. A morphological analysis of optimized film revealed a rough, particle-rich surface favorable for biomedical use. The findings highlight the promise of PHO-HAp-Curcumin composite films in advancing tissue engineering.

## 1. Introduction

In recent decades, research in materials engineering has expanded significantly, particularly studies in the field of biocompatible materials. Traditional biomaterials were made from polymers, ceramics, or metals; however, the current biomaterials incorporate therapeutic agents, including biomolecules. Currently, biomaterials represent a unique class of materials that closely interact with the human body to reproduce the functions of a tissue by regenerating it in the case of a medical condition [1,2]. They can be made from various biocompatible polymers, such as polyhydroxyalkanoates and polysaccharides, and also from non-metallic and inorganic solid materials, which constitute the class of bioceramics.

The growing world population, together with rising living standards, has created a high demand for bone replacement materials, as well as the efficiency, safety, usefulness, and durability of implants [3]. The key requirement for such materials is not to impact the host tissue.

Scaffolds used for cellular tissue growth have biocompatible and biodegradable porous structures and mechanical properties required for architecture and manufacturing technology [4].

The properties of biopolymers meet the demands for scaffold development and are safe for human use. Many research studies have produced biopolymer composite scaffolds and medical devices for tissue engineering and biomedical applications [5,6]. Polyhydroxyalkanoates (PHAs), polyesters of aliphatic hydroxy acids, have gained popularity as biodegradable polymers due to their biological (microbial) origin and non-toxicity. Polyhydroxyalkanoates are biodegradable, biocompatible, and bioresorbable, making them suitable biomaterials for medical applications such as tissue engineering, tissue reconstruction, and drug delivery systems, where they can be combined with therapeutic agents to enhance regeneration processes.

**Figure 1:**
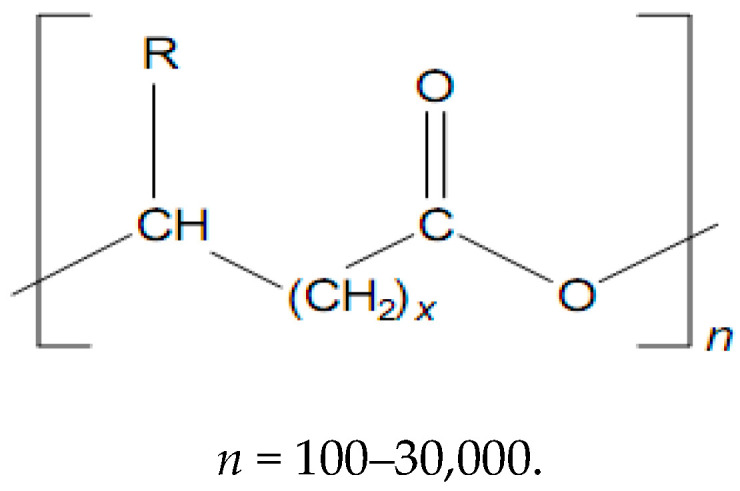
Structure of polyhydroxyalkanoates (PHAs).

The most extensively studied PHA is polyhydroxybutyrate (PHB) [7], while the most recently discovered is polyhydroxyoctanoate (PHO) [8]. PHB has been studied in biomedical applications due to its thermoplastic behavior, appropriate mechanical properties, and versatile biosintering methods [9]. Several studies have shown that medium-chain-length polyhydroxyalkanoates (*mcl*-PHAs) are much more flexible and durable than short-chain-length polyhydroxyalkanoates (*scl*-PHAs). PHA composed of monomers (Figure 1, Table 1) with 6–14 C atoms is called PHA with medium side chains, *mcl*-PHA (medium-chain-length—PHA). It has the following chemical structure, and the “R” and “*n*” are shown below:

These properties make it a viable option for use in a variety of fields, particularly medical applications and the production of films and coatings. PHA’s pleasant physical properties and high biocompatibility make it an effective raw material for the production of tablets, nanoparticles, and drug scaffolds [10]. PHAs obtained under controlled conditions and with high purity can be used in tissue engineering such as vascular grafts and nerve tissue, or as a scaffold to promote cell growth by supplying nutrition [11,12]. Kara and colleagues investigated the effects of fish scale/poly (3-hydroxybutyrate-co-3-hydroxyvalerate) (PHBV) nanofibrous composite scaffolds on bone regeneration [5]. Senatov and his collaborators also created highly porous scaffolds made of PHB and HAp to replace small bone defects in non-load-bearing parts [6]. In both cases, the effects of PHA and HAp content on the mechanical properties, thermal properties, and biodegradability of the composites were investigated.

Hydroxyapatite (HAp) is a calcium phosphate mineral that makes up a significant portion of the mineral composition of our bones and teeth. Because of its similarity to natural bone tissue, hydroxyapatite has become a reference material in bioceramics. The most notable feature of hydroxyapatite is its high biocompatibility. The human body recognizes it as its material and, in many cases, stimulates bone growth around the implant.

It has high osteoconductivity, which means that bone cells (osteoblasts) can readily adhere to its surface and form new bone. Depending on its composition and structure, it can be fully or partially resorbable, allowing for better integration with the surrounding bone [13]. One disadvantage is that it has limited mechanical strength; in some applications, pure hydroxyapatite may be insufficient, necessitating the use of other materials [14,15]. If it is synthetized at high temperatures, the mechanical strength can be slightly improved, but obtaining pure hydroxyapatite can be costly, limiting access to these materials in some areas [16].

Curcumin, a natural polyphenol extracted from *Curcuma longa*, has remarkable properties (anti-inflammatory, antimicrobial, and antioxidant activity) that make it a promising candidate for hybrid biomaterials designed to heal and prevent bone tissue diseases. To improve the efficiency of the PHO-HAp composite that we investigated for use in the treatment of joint diseases and bone regeneration, the studies presented in this paper focused on the design and optimization of curcumin-loaded polyhydroxyoctanoate (PHO) and HAp composite biomaterials [17,18,19,20].

PHO used to make the composite films was biosynthesized through microbial fermentation using the strain *Pseudomonas fluorescens* ICCF 392 from the ICCF’s Collection of Microorganisms of Industrial Importance (CMII), affiliated with the World Federation of Culture Collections (WFCC), using an original experimental model for obtaining *mcl*-PHA with controlled composition. The Design of Experiments (DoE) method was used to optimize curcumin-loaded PHO-HAp composite biomaterials for the treatment of articular diseases and bone regeneration. Specifically, the Box–Behnken experimental design, a subset of the DoE methodology, was employed to investigate the influence of PHO, HAp, and curcumin concentrations on the key properties of composite films, including biodegradability, film quality, thickness, and cumulative curcumin release. An analysis of variance (ANOVA) was used to assess the reliability and significance of the experimental model, ensuring robust predictions for optimizing film properties. The ANOVA framework enabled the identification of key interactions and effects among variables, providing insights into the relationships between composition and performance. This analysis facilitated the development of optimized PHO-HAp-Curcumin film with improved structural and functional characteristics for tissue engineering and implantable device applications.

## 2. Results and Discussions

### 2.1. Design of Experiments Studies

The design of PHO-HAp-Curcumin composite films as effective tissue engineering materials requires the consideration of biodegradability and sustained drug release. Biodegradability is crucial, as the material must degrade at a rate that aligns with tissue regeneration, facilitating seamless integration with surrounding tissue. Equally important is the controlled and sustained release of curcumin, which ensures therapeutic efficacy by maintaining optimal levels in the implanted area. A steady release promotes healing, reduces inflammation, and minimizes the risk of infection, enhancing the overall efficiency of the material. Therefore, the chosen responses for the optimization study were as follows: Y_1_—degree of biodegradability; Y_2_—film quality; Y_3_—film thickness; and Y_4_—the cumulative release of curcumin (CDR). Film quality includes film flexibility (strength), effective spreading during casting on the tray, non-sticky characteristics, the ease of peeling from the tray, and appearance. The composition of films, the quantity of polymeric material, the quantity of bioceramics, and the concentration of active substance affect their applicability in tissue engineering applications. Therefore, the independent variables selected included the amount of PHO (X_1_), the amount of HAp (X_2_), and the amount of curcumin (X_3_). These variables and their respective levels are detailed in Table 2. The optimization of the properties and composition of PHO-HAp-Curcumin films was conducted using a Box–Behnken experimental matrix comprising 3 factors, 3 levels, and 15 experiments, which included 3 replicates at the center, as presented in Table 3.

The experiments were designed in a randomized order, generated by software to minimize variation from unexpected effects in the observed responses. The BB experimental program was analyzed using the Design-Expert software, testing various models including the linear model, the 2 FI model, the quadratic model, and the cubic model. The regression model’s adequacy was assessed using ANOVA and Fisher’s F test. Interactions among variables are illustrated through contour plots (2D) and response surface curves (3D).

Table 4 presents the values of the dependent variables (Y) for each combination of independent variables (X), according to the BB experimental program.

### 2.2. Determination of the Effect of Independent Variables

#### 2.2.1. The Effect of Independent Variables on the Degree of Biodegradability (Y_1_)

The experimental data for the response Y_1_ were analyzed using the Design-Expert program. Among the tested models such as linear, 2FI, quadratic, and cubic, the 2FI model was found significantly more suitable for characterizing the relationship between the dependent and independent variables.

The analysis of variance (ANOVA) reveals an F value of 4.01 for the Fisher test, indicating that the 2FI model is statistically significant (*p* value = 0.0374 < 0.05), with only 3.74% of the total variation remaining unexplained by this model. The Adeq Precision coefficient, which quantifies the signal-to-noise ratio, is 7.6980; a number beyond 4 signifies an adequate signal. Also, the adequacy of the 2FI model is supported by the correlation coefficient, R^2^ with a value of 0.75 indicating a strong correlation, showing that 75.04% of the response variability can be explained by the model.

The simplified predictive equation, obtained by excluding the statistically insignificant terms, for the degree of biodegradability (%), is as follows:

Y_1_ = 0.7500 × X_2_ − 1.07 × X_1_X_3_ + 1.85 × X_2_X_3_ (1)

Biodegradability depended primarily on the interaction between HAp and PHO (X_2_X_3_), suggesting that the mineral phase (HAp) stabilized the matrix, slowing its breakdown. Curcumin’s influence in biodegradation is minor compared to the polymer-ceramic interplay. These findings align with studies showing that HAp enhances the stability of polymeric matrices in composites by mitigating enzymatic degradation [21].

**Figure 2:**
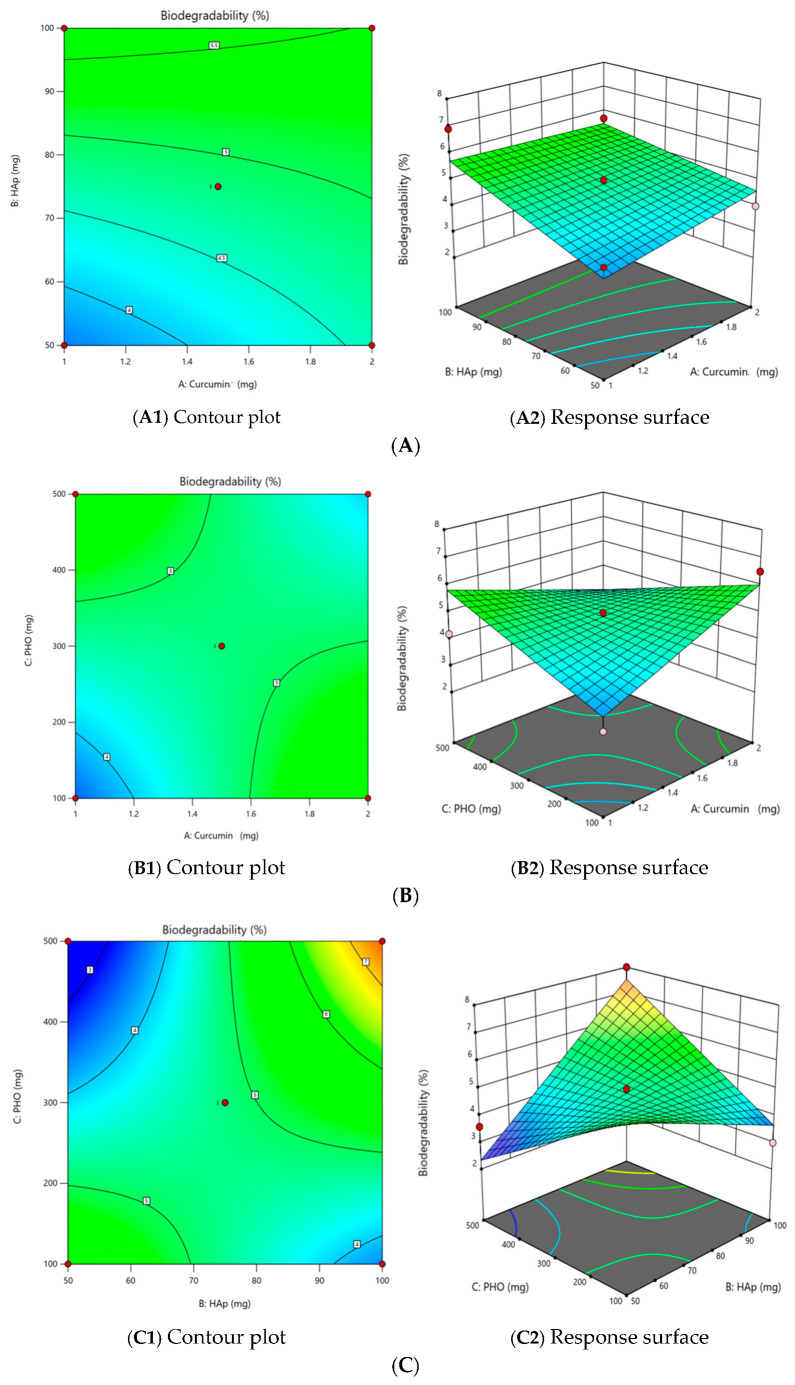
The effect of the interaction on the degree of biodegradability (Y_1_)—(**A**) between the amount of curcumin and the amount of HAp (X_1_X_2_), (**B**) between the amount of curcumin and the amount of PHO (X_1_X_3_), and (**C**) between the amount of HAp and the amount of PHO (X_2_X_3_).

The contour plots and response surfaces demonstrate that the most relevant relationship exists between HAp and PHO (X_2_X_3_) among the combinations of curcumin and HAp (X_1_X_2_), curcumin and PHO (X_1_X_3_), and HAp and PHO (X_2_X_3_) (Figure 2A–C).

#### 2.2.2. The Effect of Independent Variables on the Film Thickness (Y_3_)

The experimental data obtained for the Y_3_ response were also investigated using the Design-Expert program. Among the models tested, the quadratic model is significantly more appropriate for describing the relationship between the dependent and independent variables.

From the dispersion analysis and the ANOVA analysis of variance, it can be seen that the F value of the Fisher test is 5.76, implying that the quadratic model is statistically significant (*p* value = 0.0341 < 0.05), and only 3.41% of the total variation could not be explained by this model. In addition, the Adeq Precision coefficient, which measures the signal-to-noise ratio, has a value of 8.0815—a value greater than 4 indicates an adequate signal. The adequacy of the model is supported by the correlation coefficient (R^2^ = 0.9120), indicating a very strong correlation, with a 91.20% of the response variability explained by the model.

The simplified predictive equation for film thickness is as follows:

Y_3_ = 0.5233 − 0.1513 × X_1_ + 0.1525 × X_1_X_3_ − 0.1900 × X_2_X_3_ + 0.1996 × X_3_^2^ (2)

Curcumin and HAp likely enhance the film matrix’s density, while interactions between curcumin and PHO and HAp and PHO influenced structural integrity. Interestingly, the increase in curcumin content slightly reduced thickness beyond a threshold, possibly due to its interference with polymer. This phenomenon mirrors observations in other composite systems, where excessive active components disrupt the matrix uniformity.

**Figure 3:**
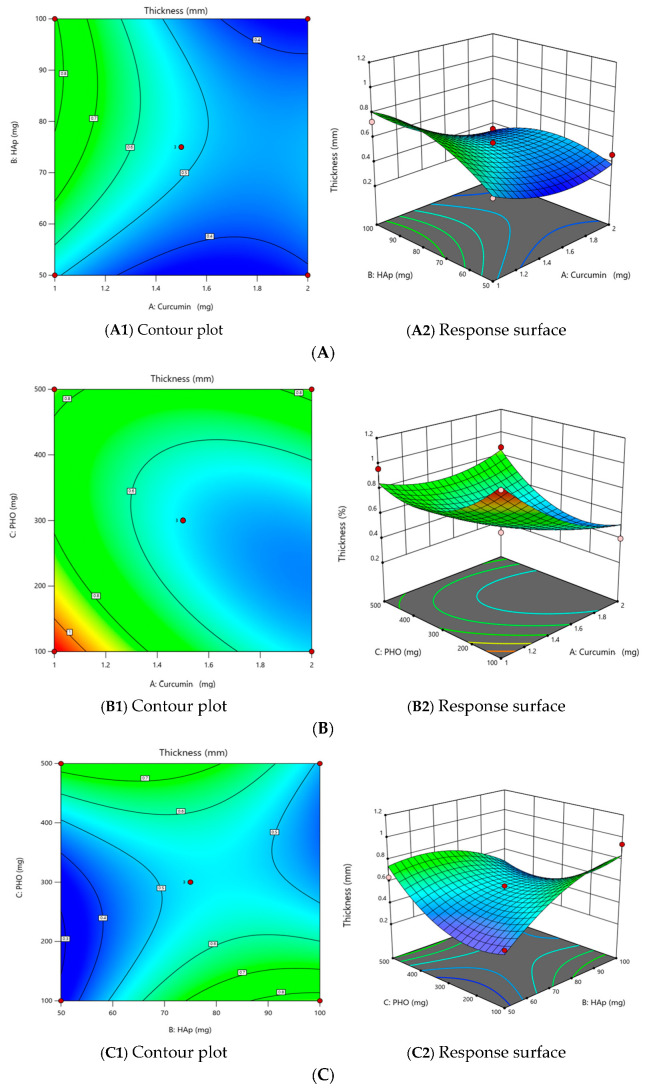
The effect of the interaction on the film thickness (Y_3_)—(**A**) between the amount of curcumin and the amount of HAp (X_1_X_2_), (**B**) between the amount of curcumin and the amount of PHO (X_1_X_3_), and (**C**) between the amount of HAp and the amount of PHO (X_2_X_3_).

The contour plots and response surfaces supported ANOVA, showing that interactions between the amount of curcumin and HAp (X_1_X_2_), the amount of curcumin and PHO (X_1_X_3_), and the amount of HAp and PHO (X_2_X_3_) are relevant for film thickness (Figure 3A–C).

#### 2.2.3. The Effect of Independent Variables on the Cumulative Release of Curcumin (CDR) (Y_4_)

The experimental data for Y_4_ were analyzed using the Design-Expert program. Among the tested models, the quadratic model is the most suitable for characterizing the relationship between the dependent and independent variables.

The dispersion analysis reveals that the Fisher test’s F value is 4.8, indicating that the quadratic model is statistically significant (*p* value = 0.0494 < 0.05), with just 4.96% of the total variation remaining unexplained by this model. Furthermore, the Adeq Precision coefficient, which quantifies the signal-to-noise ratio, is 7.0631; a value exceeding 4 signifies an adequate signal. The adequacy of the model is supported by the correlation coefficient (R^2^ = 0.8963), indicating a strong correlation.

The simplified predictive equation for CDR is as follows:

Y_4_ = −18.51 × X_1_^2^ + 14.34 × X_3_^2^ (3)

Curcumin loading (X1) contributed to a more rapid initial release, while higher PHO levels (X3) regulated the diffusion barrier, slowing the release rate. Similar trends where higher polymer content delays drug release due to reduced matrix porosity were reported [22].

**Figure 4:**
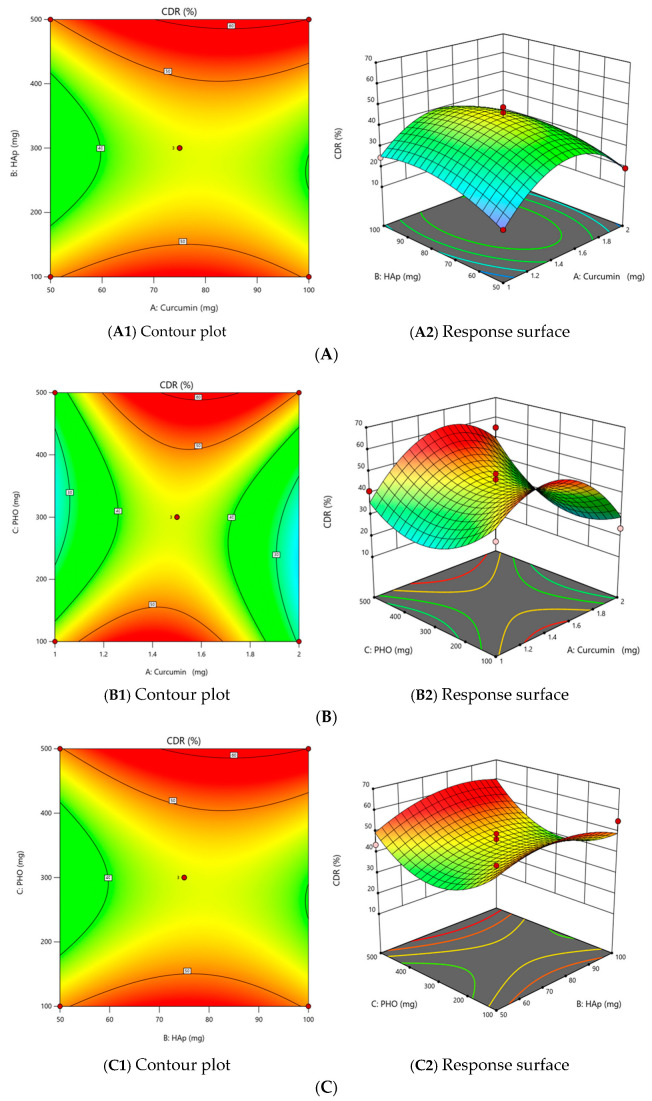
The effect of the interaction on the CDR (Y_4_)—(**A**) between the amount of curcumin and the amount of HAp (X_1_X_2_), (**B**) between the amount of curcumin and the amount of PHO (X_1_X_3_), and (**C**) between the amount of HAp and the amount of PHO (X_2_X_3_).

The contour plots and response surfaces illustrate the interactions between quantities of curcumin and HAp (X_1_X_2_), curcumin and PHO (X_1_X_3_), and HAp and PHO (X_2_X_3_) (Figure 4A–C).

#### 2.2.4. Film Quality

**Figure 5:**
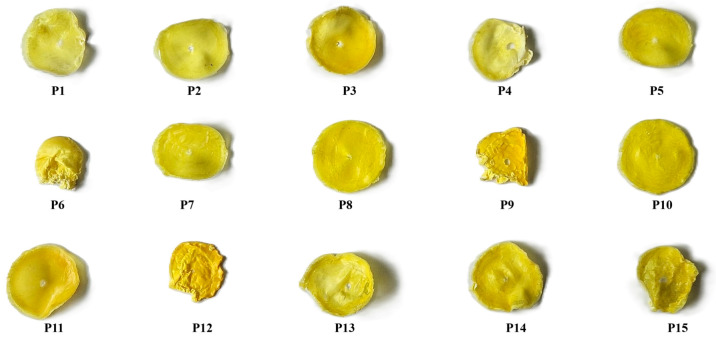
PHO-HAp-Curcumin composite polymer films.

The PHO-HAp-Curcumin composite films were evaluated using four criteria: (a) film flexibility, (b) adhesion, (c) release from the casting tray, and (d) smoothness (Table 4). Film flexibility, as measured by tensile strength, is required to withstand mechanical stress without cracking. A flexible film can better adapt to the body’s dynamic environment, lowering the likelihood of implant failure due to mechanical mismatch. Good casting spread in the casting tray refers to the ability of the film-forming solution to spread evenly across the tray, resulting in uniform film thickness and properties. Uneven spreading can cause uneven film thickness, resulting in differences in mechanical properties and biological performance. In terms of film adhesion, a non-tacky film is easier to work with during the manufacturing and implantation processes. Less sticky film reduces the risk of film damage during handling and improves manufacturing reproducibility. The release mode from the casting tray (easy detachment) simplifies the manufacturing process while lowering the risk of film deformation or damage during removal. Surface analysis evaluates the film’s appearance; thus, a smooth surface improves biocompatibility by lowering the likelihood of tissue irritation or inflammation during implantation. Surface roughness can affect cell adhesion, proliferation, and integration, so a smooth appearance is preferred for optimal tissue response. Among the PHO-HAp-Curcumin composites shown in Figure 5, samples P2, P5, P7, P8, P10, and P11 were selected as having adequate membrane quality due to their smoother appearance and adequate thickness, as indicated in Table 5.

### 2.3. Optimization of Composite Films

**Figure 6:**
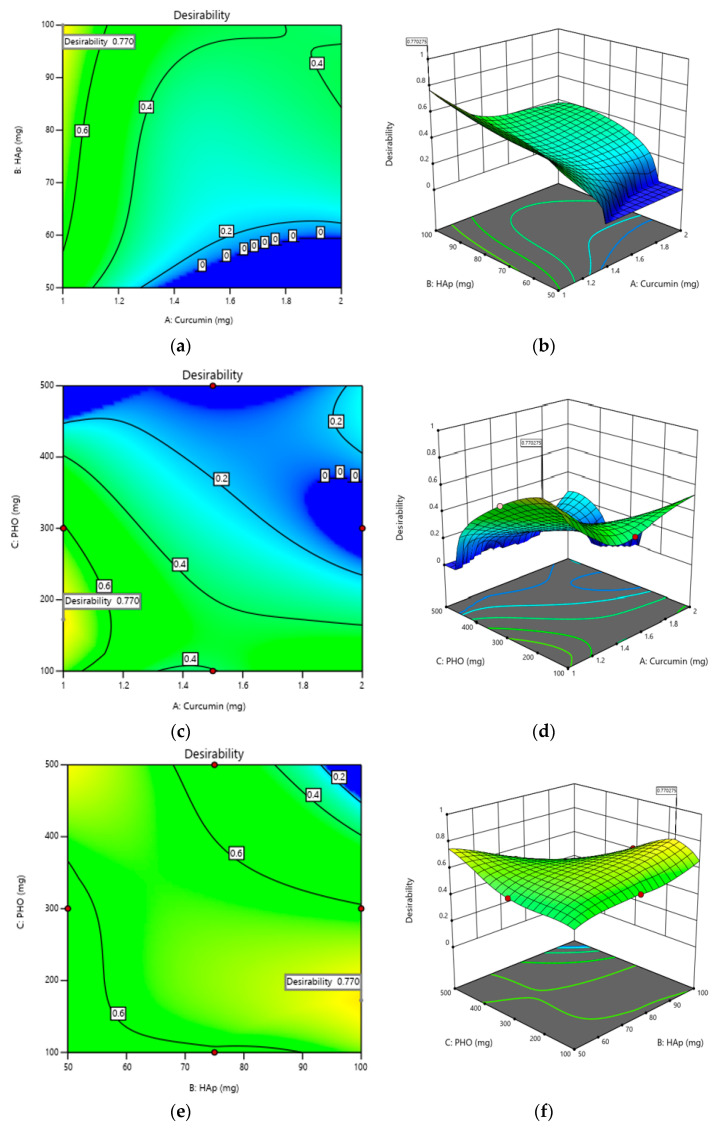
Desirability profiles—contour plots (2D) (**a**,**c**,**e**) and response surfaces (3D) (**b**,**d**,**f**).

The Design-Expert program was used to obtain the general optimum, considering the targets in Table 6. A maximum desirability of 0.777 was obtained for the optimized model variables: Curcumin amount—1 mg, HAp amount—100 mg, and PHO amount—172.309 mg, respectively. The desirability profiles are presented in Figure 6. The validation of the results were performed, the experimental results consistent with the data obtained from the desirability.

### 2.4. Characterization of the Optimum Film Obtained

After obtaining an optimum film, it was characterized from both a morphological and cytotoxic perspective.

#### 2.4.1. Morphological Evaluation

**Figure 7:**
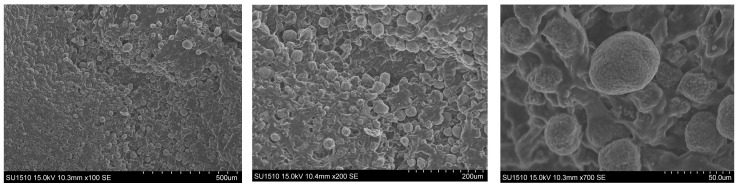
SEM images of the surface of the optimal PHO-HAp-Curcumin composite film.

The morphology of composite films was evaluated using samples with varying amounts of PHO, HAp, and Curcumin. This evaluation was performed using a microscope with two detectors: secondary electrons (SEs) and backscattered electrons (BSEs). Each produces a specific type of signal and can highlight different components depending on the structure and composition. Their variations determined the different qualities of the films, as shown by SEM images (Figure 7). SEM images revealed particles of varying sizes and shapes, possibly due to degradation during the preparation of the analyzed samples. However, the films retain the appearance and texture of the tested materials, with irregular dimensions and spaces/voids. However, the sample exhibits a contoured layer structure similar to HAp. Because the films have a unique composition, they were analyzed in comparison to the literature studies performed on polyhydroxyalkanoates [23].

#### 2.4.2. Evaluation of Cytotoxicity

**Figure 8:**
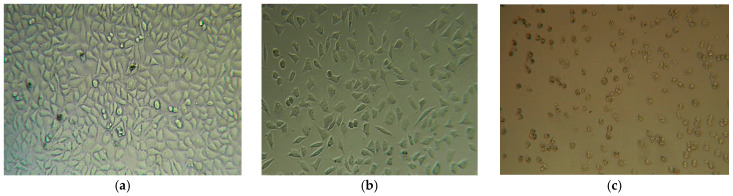
Appearance of L929 cell culture: (**a**) control, 24-well plate, (**b**) extraction method on optimum film, and (**c**) contact method on optimum film (Inverted microscope, Ob. 10×).

In compliance with regulations aimed at reducing the use of laboratory animal testing models, two sets of in vitro experiments were conducted to evaluate the cytotoxic effects of the composite film. The optimum film was tested regarding cytotoxicity using the extraction and contact methods, as recommended by the ISO 10993 standard [24,25,26]. The cytotoxicity test results indicate that the tested material is slightly cytotoxic when exposed to the test extract for 24 h, as determined using the *extract method*. Quantitative determinations are supported by microscopy images (Figure 8b), which provide visual evidence. The *contact method* classifies the sample as moderately cytotoxic after 24 h of exposure. Under 24 h exposure conditions, a morphological analysis shows that cell spreading and adhesion are inhibited, with the majority of cells rounded, detached, or with morphology changes (Figure 8c). These results suggest that residual processing solvents or unreacted curcumin may influence cytotoxicity; thus, additional purification steps or surface modifications could improve biocompatibility.

## 3. Experimental Section

### 3.1. Materials

Curcumin-loaded polyhydroxyoctanoate (PHO)–hydroxyapatite (HAp) composite films (PHO-HAp-Curcumin) were prepared using both materials produced in the laboratories of the National Institute of Chemical-Pharmaceutical Research and Development (ICCF) and commercial materials. Polyhydroxyoctanoate (PHO), a mcl-PHA polymer produced by biosynthesis [27] using the bacterial strain *Pseudomonas fluorescens* ICCF 392, from the ICCF Collection of Microorganisms of Industrial Importance (CMII), affiliated with the World Federation of Culture Collections (WFCC), was chosen as the polymer matrix of the composites.

The commercially available yellow hydroxyapatite from Sigma-Aldrich (Merck Group, Darmstadt, Germany) was used to prepare the composites. The naturally occurring mineral form of calcium apatite has the formula Ca_5_(PO_4_)_3_(OH), but it is commonly written Ca_10_(PO_4_)_6_(OH)_2_ to indicate that the crystal unit consists of two entities.

Curcumin used in the matrix of the composite films was also a pure commercial powder from Sigma-Aldrich. Curcumin, C_21_H_20_O_6_, has two aromatic phenolic rings linked by two α,β-unsaturated carbonyl groups. The diketone group produces a stable enol, which is easily deprotonated to yield an enolate ion. Carbonyl groups are Michael acceptors that undergo nucleophilic addition reactions. Curcumin is a diarylheptanoid and a member of the curcuminoid class. It is a compound that exhibits keto-enolic tautomerism (it exists in both enolic and ketone forms when dissolved in organic solvents and water).

### 3.2. Methods

#### 3.2.1. The Preparation of Composite Films

**Figure 9:**
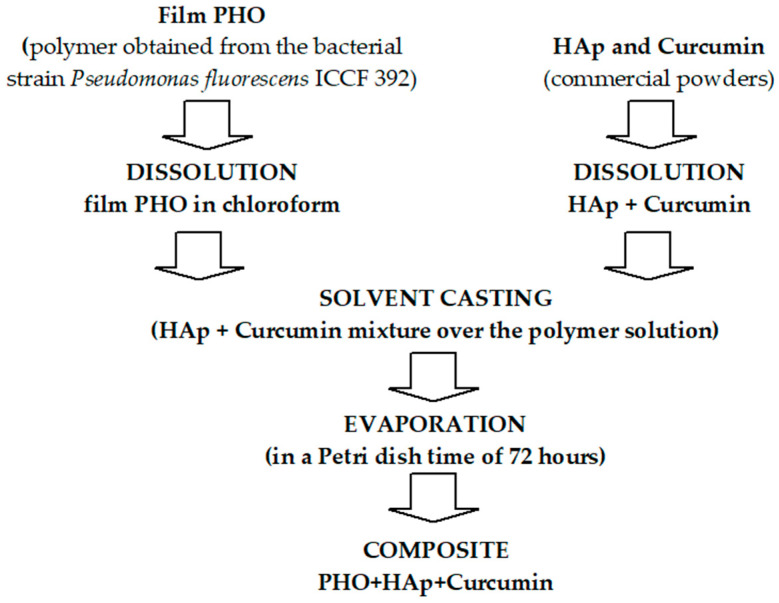
Preparation scheme of PHO-Hap-Curcumin composite films.

To conduct optimization studies, polymer–ceramic–therapeutic agent (PHO-HAp-Curcumin) composite films were prepared using the solvent casting method (Figure 9). The polymer, ceramic, and active substances were weighed in accordance with Table 2. PHO was dissolved in chloroform under magnetic stirring, and Hap and curcumin were added to the polymer mixture. The resulting suspensions were poured into Petri dishes with diameters of 250 mm and allowed to dry at room temperature for 72 h to fully evaporate the solvent. The films were carefully removed from the Petri dish’s surface and stored in a dry place for future analyses (Figure 5).

#### 3.2.2. Characterization of Composite Films

##### Design of Experiment

The Design-Expert Program (Stat-Ease 360-Trial Version, Stat-Ease Inc., Minneapolis, HE, USA) was used to validate the development of PHO-HAp-Curcumin composite films. This program allowed for the understanding of the impact of independent variables (amounts of PHO, HAp, and curcumin, respectively) on the degree of biodegradability, film thickness, and curcumin release (dependable variables).

##### Evaluation of Film Thickness

Three films per formulation were used to determine thickness. Each was measured at 3 points (2 corners and the center) with a Digital Display Stainless Steel Caliper (TESA Technology, Renens, Switzerland), and the average value was taken.

##### Biodegradability Evaluation

Biodegradability was assessed by monitoring weight loss at different time intervals. Composites were cut to 0.5 × 0.5 cm^2^, weighed accurately, and then placed in 15 mL plastic tubes containing 8 mL of a body fluid simulating solution, SBF (1 L of SBF solution contains chloride of sodium—7.996 g, sodium bicarbonate—0.350 g, potassium chloride—0.224 g, dibasic potassium phosphate (K_2_HPO_4_ × 3H_2_O)—0.228 g, magnesium chloride (MgCl_2_ × 6H_2_O)—0.305 g, 1 M of hydrochloric acid—40 mL, calcium chloride—0.278 g, sodium sulfate—0.071 g, tris(hydroxymethyl) aminomethane—6.057 g and ultrapure water—740 mL), with a pH of 7.2. These tubes were then placed in a shaking incubator set at 100 rpm and 37 °C (Memmert GmbH Model M00 water bath, Schwabach, Germany). At a predetermined interval (one week), the composites were removed and dried in a desiccator to constant weight and weighed three times, resulting in an average value. The total biodegradability assessment period was 3 weeks.

The percent weight loss (%) was calculated using the following formula:

(4) $$Weight\;loss\;(\%)=\frac{Wi-Wf}{Wi}\times 100$$

where *Wi* and *Wf* represent the initial weight of the sample and the final weight after biodegradation, respectively.

##### Evaluation of Cumulative Release of Curcumin (CDR)

For the evaluation of in vitro curcumin release (CDR), a 0.5 × 0.5 cm^2^ film sample was immersed in a 40 mL ethanolic solution (30%) while maintaining perfect immersion conditions. The samples were kept at a temperature of 37 °C, under continuous agitation (100 rpm/min) with the addition of the release medium, by maintaining the same immersion volume. The released active substance was determined spectrophotometrically using a UV–VIS spectrophotometer (JASCO V-630 Spectrophotometer, Jasco International Co., Ltd., Tokyo, Japan). The calibration curve of curcumin in ethanol (30%) was used to determine the yielded active substances. The selection of a 30% ethanol solution as a release medium for the assessment of the cumulative drug release (CDR) of curcumin was influenced by the need to adequately dissolve curcumin, a highly hydrophobic compound and facilitate accurate measurement using UV–Vis spectrophotometry. This method is used for hydrophobic substances in the scientific literature due to its reproducibility and sensitivity in in vitro studies. Hamilton et al. (2023) provide an overview of curcumin delivery mechanisms, including the use of ethanol as a release medium [20].

##### Evaluation of the Cytotoxic Effects

The extraction method and the contact method, which are the methodologies suggested by the ISO 10993 standard [24,25,26], were used to conduct the testing of cytotoxic effects on L-929 mouse fibroblasts cells (ATCC^®^CRL-6364, Manassas, VA, USA). The qualitative evaluation of cytotoxic effects was performed by examining cell morphology, the degree of spreading, vascularization and detachment, cell lysis, and membrane integrity. Quantitative cytotoxic effects were evaluated by determining cell viability by the MTS method using the CellTiter96^®^AQueous-Non-Radioactive-Cell-Proliferation-Assay (Promega, Madison, WI, USA).

## 4. Conclusions

These studies conducted to develop PHO-HAp-Curcumin composite films were validated using the Design-Expert Program (State Ease Trial), which allowed an understanding of the effect of the independent variables (amounts of PHO, HAp, and Curcumin, respectively) on the degree of biodegradability, film thickness, and Curcumin release. Statistical modeling revealed significant interactions among the components, with the 2FI and quadratic models providing strong predictive accuracy. Thus, when compared to other combinations of variables, the interaction of HAp and PHO (X_2_X_3_) has a significant influence on biodegradability (Y_1_) and film thickness (Y_3_). There was no significant interaction between the independent variables (curcumin-X_1_, HAp-X_2_, and PHO-X_3_) in terms of the degree of release (CDR). The ANOVA analysis supports the conclusions about the interactions, demonstrating the model’s reliability. The optimized film exhibited a maximum desirability of 0.777 with 1 mg of curcumin, 100 mg of HAp, and 172.31 mg of PHO. Morphological analysis of the optimized film revealed a rough, particle-rich surface favorable for biomedical use. Preliminary results of in vitro biocompatibility evaluation reveal that the tested samples present different degrees of cytotoxicity on L929 cells, which conditions their use for in vivo applications. Considering that this research presented was completed by obtaining composite films with applications in tissue engineering, it would be necessary to continue the in vivo investigation on specific cell lines to finalize their characterization. Determining the long-term stability of the materials produced would be an opportunity for the development of pharmaceutical biotechnology. Starting with this concept, future research should concentrate on completing the characterization of pharmaceutical materials, which requires a set of specific techniques. Future research can focus on determinations using thermal analysis methods such as differential scanning calorimetry, thermogravimetry, differential thermal analysis, and thermomicroscopy.

## Figures and Tables

**Table 1:** PHAs types.

*x*	*R*	*Polymer Name*
1	H	Poly-(3-hydroxypropionate)
	CH_3_–	Poly-(3-hydroxybutyrate)
	CH_3_–CH_2_–	Poly-(3-hydroxyvalerate)
	CH_3_–CH_2_–CH_2_–	Poly-(3-hydroxyhexanoate)
	CH_3_–(CH_2_)_3_–CH_2_–	Poly-(3-hydroxyoctanoate)
	CH_3_–(CH_2_)_7_–CH_2_–	Poly-(3-hydroxydodecanoate)
2	H	Poly-(4-hydroxybutyrate)
3	H	Poly-(5-hydroxyvalerate)

**Table 2:** Independent variables and experimental levels.

Independent Variables.	Level		
	−1	0	1
The amount of curcumin (X_1_), mg	1	1.5	2
The amount of HAp (X_2_), mg	50	75	100
The amount of PHO (X_3_), mg	100	300	500

**Table 3:** Box–Behnken experimental matrix of independent variables.

Std	Run	Level X_1_	Level X_2_	Level X_3_	X_1_ (mg)	X_2_ (mg)	X_3_ (mg)
P1	2	−1	−1	0	1	50	300
P2	7	−1	1	0	2	50	300
P3	6	1	1	0	1	100	300
P4	11	0	−1	−1	2	100	300
P5	13	0	−1	1	1	75	100
P6	1	−1	0	−1	2	75	100
P7	8	−1	0	1	1	75	500
P8	9	1	0	1	2	75	500
P9	10	0	1	−1	1.5	50	100
P10	14	0	1	1	1.5	100	100
P11	3	1	−1	0	1.5	50	500
P12	5	1	0	−1	1.5	100	500
P13	4	0	0	0	1.5	75	300
P14	15	0	0	0	1.5	75	300
P15	12	0	0	0	1.5	75	300

**Table 4:** The answers obtained: the dependent variables (Y) for each combination of independent variables (X).

Std	Run	X_1_ (mg)	X_2_ (mg)	X_3_ (mg)	Y_1_ Biodegradability (%)	Y_2_ Film Quality					Y_3_ Thickness (mm)	Y_4_ CDR (%)
						Flexibility (%)	Adhesion (%)	Peeling Mode (%)	Smoothing (%)	Total Film Quality (%)		
P1	2	1	50	300	4	25	12.5	25	25	87.5	0.5	14.4
**P2**	**7**	**2**	**50**	**300**	4	**25**	**25**	**25**	**25**	**100**	0.46	19.3
P3	6	1	100	300	6.9	25	12.5	25	25	87.5	0.73	24.6
P4	11	2	100	300	5.7	25	12.5	25	12.5	75	0.35	18
**P5**	**13**	**1**	**75**	**100**	3	**25**	**25**	**25**	**25**	**100**	1.1	39.8
P6	1	2	75	100	6.5	25	12.5	12.5	0	50	0.4	23.7
**P7**	**8**	**1**	**75**	**500**	4.2	**25**	**25**	**25**	**25**	**100**	0.96	41.1
**P8**	**9**	**2**	**75**	**500**	3.4	**25**	**25**	**25**	**25**	**100**	0.87	55
P9	10	1.5	50	100	6	25	12.5	12.5	12.5	62.5	0.37	53.9
**P10**	**14**	**1.5**	**100**	**100**	3	**25**	**25**	**25**	**25**	**100**	0.94	55
**P11**	**3**	**1.5**	**50**	**500**	3.6	**25**	**25**	**25**	**25**	**100**	0.64	43.8
P12	5	1.5	100	500	8	25	12.5	12.5	0	50	0.45	55
P13	4	1.5	75	300	4.7	25	12.5	25	12.5	75	0.56	46.5
P14	15	1.5	75	300	5	25	12.5	25	12.5	75	0.56	49.1
P15	12	1.5	75	300	4.7	25	12.5	12.5	12.5	62.5	0.45	36.6

**Table 5:** Criteria for film quality highlighting the distribution of scores.

Characteristic	Score (%)		
	Poor Score (0%)	Average Score (12.5%)	Good Score (25%)
The flexibility of the film	Resistance < 20 ori	Resistance 20–30	Resistance > 30 ori
Adhesion	Very sticky film, cannot be processed easily	Slightly sticky but could be easily worked	Non-sticky, processable film
Peeling mode	Very adherent	Slightly adherent	Non-adherent
Smoothing	The surface is not smooth, with irregularities or voids	Smooth surface with some irregularities	Smooth surface

**Table 6:** Response targets for achieving the overall optimum.

Answer	Aim	Justification
Y_1_—The degree of biodegradability	Minimize	Minimizing the degree of biodegradation to maintain the structural integrity of the composite films over time, thus to ensure their long-term stability and durability in the biological environment.
Y_3_—Film thickness	Maximize	A higher film thickness will imply better film quality and improved mechanical properties.
Y_4_—CDR	Minimize	Minimizing the cumulative release of curcumin will lead to the efficient release of curcumin in a controlled and sustained manner at the implant site.

## Data Availability

Data to support statements in this article are available from the corresponding author [Dana-Maria Miu], upon reasonable request.
